# Transcriptomic Indicators of the Point of No Return in *Leiocassis longirostris*: Ecological Thresholds and Molecular Signatures of Failed Nutritional Transition

**DOI:** 10.1002/ece3.74726

**Published:** 2026-11-14

**Authors:** Shanshan Guan, Xiangchao Si, Jinhui Yu, Jianfang Guo, Ruixuan Qi, Meng Yuan, Yongtao Tang, Qiang Li, Chuanjiang Zhou

**Affiliations:** ^1^ The Observation and Research Field Station of Taihang Mountain Forest Ecosystems of Henan Province, College of Life Sciences Henan Normal University Xinxiang People's Republic of China; ^2^ College of Fisheries Henan Normal University Xinxiang Henan People's Republic of China; ^3^ Fisheries Institute Sichuan Academy of Agricultural Sciences Chengdu People's Republic of China

**Keywords:** *Leiocassis longirostris*, nutritional transition, point of no return (PNR), transcriptomic analysis

## Abstract

The transition from endogenous yolk utilization to exogenous feeding is a critical developmental event in larval fish. Failure to complete this transition during starvation may lead to the point of no return (PNR). We conducted transcriptomic sequencing at eight developmental stages (F2, F3, F5, S5, F9, S9, F14, and S14/PNR) in *Leiocassis longirostris* larvae to investigate the molecular mechanisms underlying nutritional transition and starvation‐induced PNR. During normal development, the transition to the exogenous nutrition phase was characterized by a coordinated program encompassing translational activation, immune‐barrier establishment, and enhanced nutrient utilization. In contrast, under starvation, larvae exhibited a progressive trajectory from adaptive compensation to systemic failure. Early starvation was marked by PPAR‐mediated lipid mobilization and cell‐cycle suppression to conserve energy. Upon reaching the PNR, the transcriptomic profile shifted toward terminal catabolism, characterized by significant proteasome activation and the catastrophic disruption of ECM–receptor interactions and focal adhesions, signaling a loss of tissue integrity. WGCNA identified a PNR‐specific module enriched in programmed cell death and inflammatory signaling. We propose a mechanistic model for the PNR driven by necroptosis via the RIPK1–RIPK3–MLKL axis and persistent inflammation involving p65 and STAT3/6. Cross‐sectional comparisons of the starvation and feeding groups revealed a progression from early adaptive metabolic signaling to terminal bioenergetic collapse and irreversible structural degradation. The PNR therefore represents a critical ecological threshold, with the identified pathways and genes offering candidate indicators of larval feeding success and survival potential.

## Graphical abstract

This study systematically characterized the transcriptomic landscape of *Leiocassis longirostris* larvae during the nutritional transition and progressive starvation leading to the point of no return (PNR). We identified a hierarchical progression from early adaptive metabolic compensation to terminal bioenergetic collapse and irreversible structural degradation driven by necroptosis and chronic inflammation. These findings provide critical molecular indicators for assessing larval survival potential and optimizing management strategies in aquaculture.

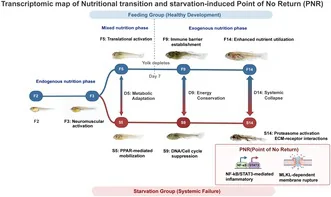

## 1 Introduction

The long‐snouted catfish ( *Leiocassis longirostris* ), commonly referred to as Jiangtuan or Feiyu, is a unique freshwater economic fish species endemic to China. It belongs to the family Bagridae within the order Siluriformes. This species occurs mainly in the main stem of the Yangtze River and its large connected lakes, as well as the Pearl River and Heilongjiang River systems (Duan et al. 2025). Following the first successful artificial breeding of this species by the Sichuan Yangtze River Aquatic Resources Survey Team in 1974, research on its aquaculture methods has continued for over four decades. However, larval mortality remains high. This is largely attributed to difficulties in the transition from yolk‐dependent endogenous nutrition to autonomous exogenous feeding. This bottleneck significantly hinders seed production and the expansion of aquaculture scale (Guo et al. 2025). The larval development of *L. longirostris* strictly follows a well‐defined nutritional sequence. During the first 4 days after hatching, larvae depend exclusively on yolk reserves. Around Day 4, the mixed nutrition phase begins with first feeding. Yolk reserves are generally depleted by Day 7, signifying the transition to complete exogenous nutrition. If larvae fail to begin feeding, prolonged starvation leads to the PNR. Under such conditions, although some individuals survive, more than 50% of larvae lose the ability to feed, which indicates that they have reached a state of ecological death. In *L. longirostris* , this threshold occurs on Day 14 post‐hatching under standard rearing conditions, as empirically determined through starvation‐refeeding experiments in our previous study (Xu et al. 2025). In natural environments, failure to initiate exogenous feeding promptly or to obtain suitable prey may similarly trigger PNR, making this a biologically and ecologically relevant threshold for assessing larval survival. Under starvation, while PNR‐induced systemic catabolism and failure of organogenesis are conserved across teleosts, the latency of this threshold follows different ontogenetic trajectories. Fast‐developing species, such as silver perch ( *Leiopotherapon plumbeus* ), reach this threshold rapidly within 48–96 h after hatching. In contrast, cold‐water species, including Chinese sturgeon ( *Acipenser sinensis* ) and walleye pollock ( *Gadus chalcogrammus* ), can maintain viability for at least 14 days (Chai et al. 2014; Garcia et al. 2020; Yokota et al. 2016). These species‐specific differences highlight the need for targeted investigations into the underlying molecular mechanisms governing the PNR stage in economically important species like *L. longirostris* . Although researchers have extensively studied the reproductive biology, aquaculture techniques, and nutritional requirements of *L. longirostris* (Tan et al. 2007), and even performed whole‐genome sequencing‐based comparative genomic analysis related to its feeding preference and metabolic traits (Mou et al. 2022; Ye et al. 2023), the exact molecular regulatory mechanisms underlying larval nutritional transformation and the physiological responses to prolonged starvation, especially those leading to the PNR, need to be elucidated. This knowledge gap limits our understanding of the molecular networks underlying nutritional adaptation and starvation responses in *L. longirostris* larvae, and limits the identification of developmental checkpoints and early indicators of starvation‐induced damage. Although studies on other teleosts have found that starvation activates oxidative stress and homeostatic pathways such as FoxO and p53 (Feng et al. 2025; Han et al. 2023), the relevance of these findings to larval development in *L. longirostris* remains unclear. To address these challenges, we performed high‐throughput RNA sequencing to systematically characterize transcriptomic changes in larvae during the critical period of nutritional transition and throughout the process of progressive starvation, to clarify the underlying molecular mechanisms (Fu et al. 2019; Qi et al. 2023). This study was designed with the following objectives:

1. To map the core regulatory pathways that drive the transition from endogenous to exogenous nutrition.
2. To describe the molecular progression from initial reserve mobilization to systemic collapse at the PNR stage.
3. To identify candidate molecular indicators associated with feeding success, survival potential, and recruitment risk in *L. longirostris* . Through integrated longitudinal and cross‐sectional analyses, this study provides a mechanistic and ecological framework for optimizing larval rearing protocols and evaluating survival thresholds in both cultured and wild populations.

## 2 Materials and Methods

### 2.1 Experimental Animals

In May 2024, healthy larvae of *L. longirostris* were obtained from the Qianwei Branch of the Minjiang Port and Shipping Electric Power Development Company (Leshan, China). Larvae were reared in an indoor recirculating aquaculture system, with each experimental group housed in a separate tank with continuous water circulation to maintain water quality. Water temperature was maintained at 23.5°C–26.5°C. Larvae in the feeding group received water fleas from the onset of mouth opening (4 dph) until the end of the experiment, whereas the starvation group was not fed. The rearing protocol and measurements of body length across developmental stages followed those described in our previous study (Xu et al. 2025).

### 2.2 RNA Sampling, Extraction, Quantification, and Qualification

To characterize the transcriptomic response of *L. longirostris* to nutritional transition and starvation, samples were collected from the feeding (F) and starvation (S) groups at five developmental time points: Day 2 (F2), Day 3 (F3), Day 5 (F5, S5), Day 9 (F9, S9), and Day 14 (F14, S14) (Figure 1). Day 2 (F2) and Day 3 (F3) represent the yolk‐dependent endogenous nutrition phase. Day 5 (F5, S5) corresponds to the mixed nutrition phase, characterized by partial yolk depletion and the onset of feeding. Day 9 (F9, S9) marks the fully exogenous nutrition phase, when yolk reserves are depleted. Day 14 (F14, S14) corresponds to the PNR stage for the starvation group, as previously defined by the inability of more than 50% of larvae to resume feeding after food was restored. At each sampling time point, 20 larvae were randomly selected from each group. After excess surface moisture was removed from the specimens with sterile absorbent paper, the specimens were immediately immersed in RNAlater (Ambion). The samples were equilibrated at 4°C overnight to allow complete tissue penetration, and were then transferred to −80°C for long‐term storage. For transcriptomic analysis, three biological replicates were set up at each time point. Each replicate consisted of a pool of five larvae (*n* = 15 larvae per group/time point) to minimize biological variation.

**FIGURE 1:**
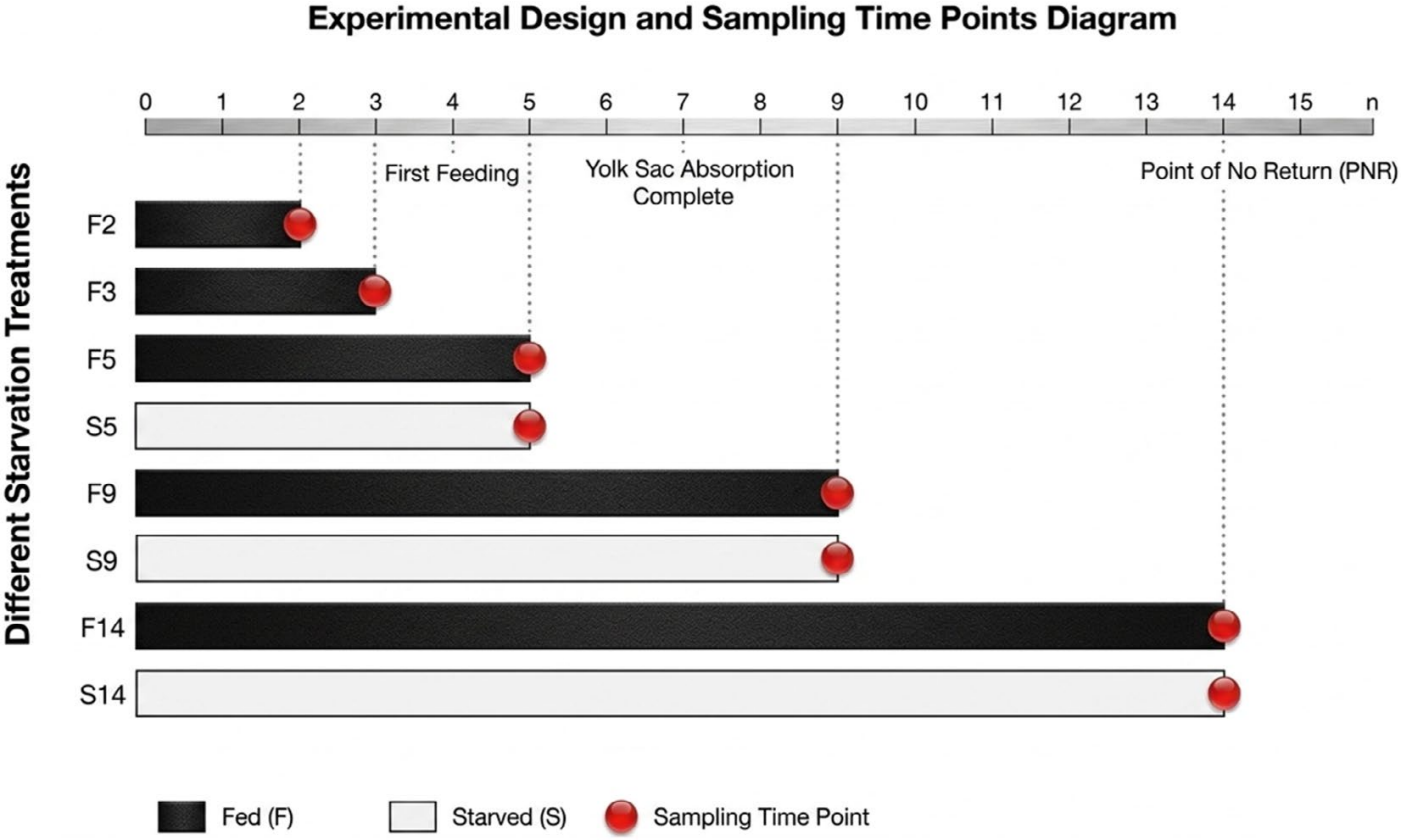
Experimental design and sampling time points.

Total RNA was extracted from larval samples using TRIzol Reagent (Life Technologies, California, USA) following the manufacturer's instructions. RNA concentration and purity were determined using a NanoDrop 2000 (Thermo Fisher Scientific, Wilmington, DE). The integrity of the RNA was evaluated using an RNA Nano 6000 Assay Kit and an Agilent Bioanalyzer 2100 system (Agilent Technologies, CA, USA) (Xu et al. 2022).

### 2.3 Library Preparation for Transcriptome Sequencing

For each sample, 1 μg of total RNA was used as the input material. Sequencing libraries were prepared using the Hieff NGS Ultima Dual‐mode mRNA Library Prep Kit for Illumina (Yeasen Biotechnology, Shanghai, China) following the manufacturer's instructions. Briefly, poly‐A mRNA was enriched using oligo(dT) magnetic beads and subsequently fragmented. First‐strand and second‐strand cDNA were synthesized, followed by end‐repair, 3′ adenylation, and ligation of hairpin‐loop adapters. After size selection was performed using the AMPure XP system (Beckman Coulter, CA, USA), the cDNA libraries were amplified by conducting polymerase chain reaction (PCR) using Phusion High‐Fidelity DNA polymerase. The quality of the libraries and the size of the insert were evaluated using the Agilent Bioanalyzer 2100 system.

### 2.4 Clustering and Sequencing

After library quality control was completed, different libraries were pooled based on the effective concentration and targeted data amount. Subsequently, the pooled libraries were sequenced on an Illumina NovaSeq 6000 platform, generating 150 bp paired‐end reads according to the manufacturer's instructions. The raw sequencing data have been deposited in the NCBI Sequence Read Archive (SRA) database under the BioProject accession number PRJNA1445138.

### 2.5 Sequencing Data Quality Control and Analysis

Raw data in the fastq format were initially processed using the fastp software (Chen et al. 2018; Peter et al. 2010). In this step, clean data were obtained by eliminating reads containing adapters, reads containing poly‐N, and low‐quality reads from the raw data. Simultaneously, the Q20, Q30, and GC content of the clean data were computed. All subsequent analyses were performed using these high‐quality clean data.

Reference genome sequences and the corresponding gene annotation files were downloaded from the genome database. The reference genome was identified as *Leiocassis‐longirostris*. Customer‐V1.genome.fa (He et al. 2021). HISAT2 (version 2.0.4) was used to align clean reads with the reference genome (Kim et al. 2015), and mapping information was obtained. StringTie (version v2.2.1) was subsequently used to assemble the aligned reads and reconstruct the transcriptome for subsequent analysis (Pertea et al. 2015). Transcript abundance was quantified using the maximum‐flow algorithm in StringTie and normalized as Fragments Per Kilobase of transcript per Million mapped fragments (FPKM) (Trapnell et al. 2010). This normalization strategy was selected because it accounts for sequencing depth and gene length, providing a standardized measure that minimizes technical bias across the transition from endogenous to exogenous feeding. To assess the quality and reproducibility of the RNA‐seq data, FPKM distributions were examined across all samples.

### 2.6 Screening of DEGs

Differentially expressed genes (DEGs) were identified using the DESeq2 (version 1.30.1) package, using screening criteria of |log_2_FC (Fold Change)| ≥ 1 and false discovery rate (FDR) < 0.05. The fold‐change cutoff was selected to identify biologically meaningful expression changes, while the FDR threshold accounted for multiple testing and limited the expected proportion of false positives. This combined criterion is widely used in transcriptomic studies and balances stringency with sensitivity in detecting robust transcriptional responses. To capture the molecular dynamics of development and starvation, we established three comparative dimensions as follows:

1. Ontogenetic transitions: Longitudinal analyses compared F3 with F2 (endogenous phase), F5 with F3 (mixed nutrition phase), F9 with F5 (early exogenous phase), and F14 with F9 (stable exogenous nutrition).
2. Progression of starvation: Sequential comparisons were conducted between S5 and F3 (yolk consumption), S9 and S5 (transition to endogenous tissue catabolism), and S14 and S9 (transition to the PNR stage).
3. Cross‐sectional comparison between the starvation and feeding groups: Direct comparisons were performed between the starvation and feeding groups at corresponding time points (S5 vs. F5, S9 vs. F9, and S14 vs. F14).

### 2.7 GO, KEGG Pathway Enrichment Analysis, and WGCNA

Gene ontology (GO) functional annotation and enrichment analysis were performed using InterProScan (version 5.34‐73.0) with reference to the GO database (http://www.geneontology.org/). The selected DEGs were annotated and categorized into the three principal GO categories, which included biological process (BP), cellular component (CC), and molecular function (MF). A *q*‐value threshold below 0.05 was applied to identify GO entries that were significantly enriched (Ashburner et al. 2000). The KEGG database (http://www.genome.jp/kegg/), with the same q‐value threshold of less than 0.05, was used to identify significantly enriched Kyoto Encyclopedia of Genes and Genomes (KEGG) pathways (Minoru et al. 2004). This approach comprehensively lists the enriched pathways across all comparison groups, revealing the main physiological and metabolic processes associated with the DEGs.

Weighted gene co‐expression network analysis (WGCNA) was performed using the WGCNA package in the R programming environment. Genes with expression levels > 1 were retained for network construction. The soft‐thresholding power was determined to attain a scale‐free network. The minimum module size was set at 30, and the module similarity threshold was set to 0.25. Sample analyses were not merged. Gene modules were identified based on topological overlap, and correlations between modules and traits were then calculated to determine the modules most strongly associated with the phenotype of interest. Modules associated with starvation stress and the PNR stage were identified. Using module–phenotype association analysis, we selected key modules exhibiting a significant positive correlation with S14 (PNR stage). The sequences were searched using BLASTX against the genome of a related species (for which protein–protein interaction (PPI) data were available in the STRING database: http://string‐db.org/) to obtain predicted PPIs for these sequences. The resulting PPI network was then visualized in Cytoscape to identify core regulatory genes (Franceschini et al. 2012).

### 2.8 Real‐Time Quantitative PCR (qRT‐PCR) Verification

To assess the reliability of the transcriptome sequencing results, seven DEGs (*rpl35* (*60S ribosomal protein L35*), *ndufa1* (*NADH dehydrogenase [ubiquinone] 1 alpha subcomplex subunit 1*), *socs3a* (*suppressor of cytokine signaling 3a*), *nfkbiaa* (*nuclear factor of kappa light polypeptide gene enhancer in B‐cells inhibitor, alpha a*), *stat3* (*signal transducer and activator of transcription 3 isoform X1*), *traf3* (*TNF receptor‐associated factor 3*), *and Llo_GLEAN_10021876* (*transcription factor NFkB p65 subunit*)) were selected for validation by qRT‐PCR. Actin, a commonly used housekeeping gene that is stably expressed in most cells, was used to correct the relative expression levels of mRNA, thereby reducing between‐sample variation and improving measurement accuracy. Primers were designed using the Primer 5 software (Supplementary Table 1). Supplementary Table 1 lists all primer pairs used in this study, including the full gene names, forward and reverse primer sequences (5′‐3′). The qRT‐PCR reaction system (10 μL) consisted of 5 μL of 2× ChamQ Universal SYBR qPCR Master Mix, 0.2 μL each of forward/reverse primers (10 μM), 1 μL of cDNA template, and dd H_2_O to make up to 10 μL. The thermal cycling program was as follows: 95°C pre‐denaturation for 30 s; 40 cycles of 95°C for 10 s and 60°C for 30 s. Melting curve analysis was performed to verify the specificity of amplification products. Relative gene expression was calculated using the 2−ΔΔCₜ method. To assess expression levels, three biological and three technical replicates were included.

## 3 Results

### 3.1 Sequencing Data Statistics

After quality filtering, 188.30 Gb of clean sequencing data were generated, with Q30 values ≥ 96.87% across all samples, indicating that the sequencing results were of high quality (Supplementary Table 2) (Kruglyak et al. 2024), Supplementary Table 2 also shows high Q30 scores and consistent GC content across all samples.

### 3.2 Statistical Analysis of Alignment Rates Between Sequencing Data and Reference Genomes and Quality Assessment

The sequencing data were highly reliable, with mapping rates against the *L. longirostris* reference genome ranging from 90.49% to 95.23%. These rates exceeded the standard threshold for transcriptomic profiling, providing a robust foundation for subsequent bioinformatic analyses (Sims et al. 2014). PCA and FPKM distributions also indicated good reproducibility among biological replicates and distinct clustering according to developmental stage and nutritional condition (Figure 2).

**FIGURE 2:**
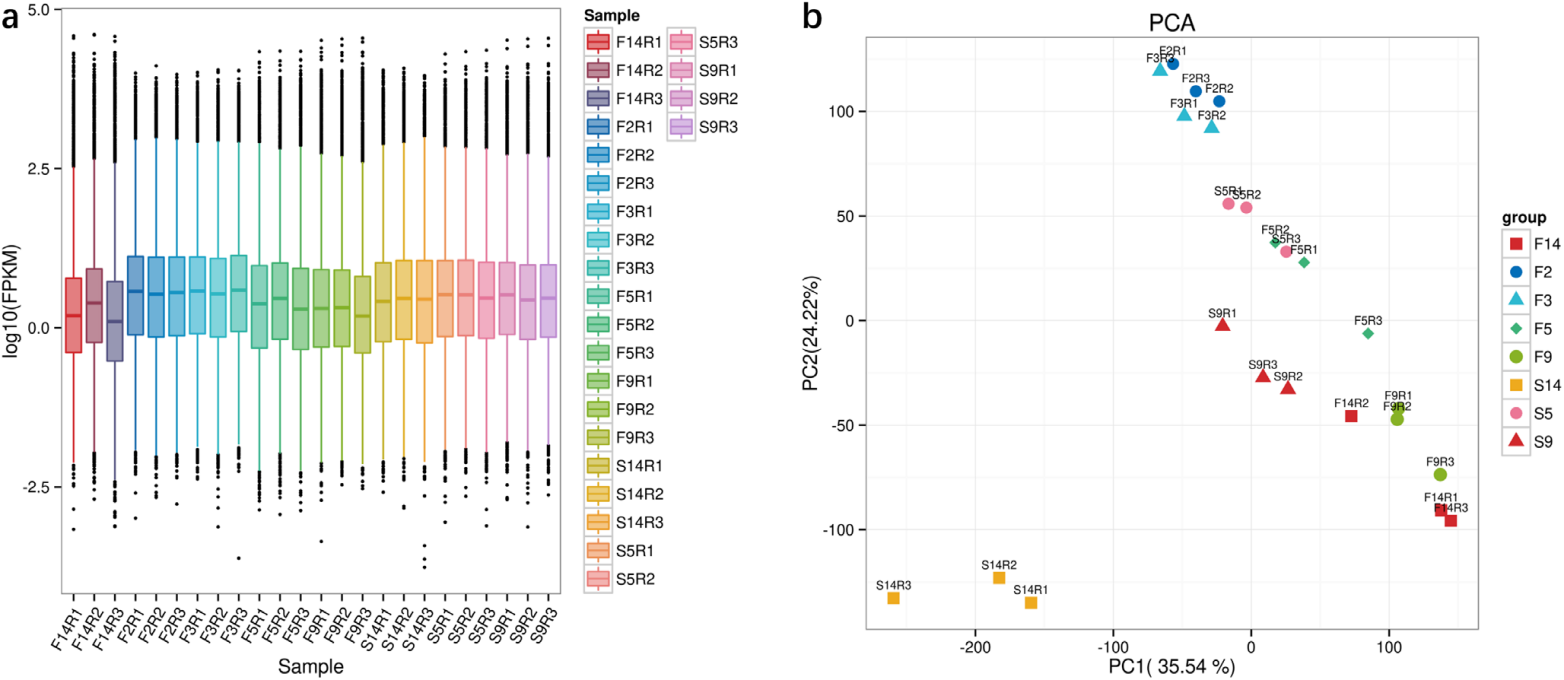
Quality Assessment of RNA‐seq Data based on FPKM Distribution and PCA. (a) Boxplots show the distribution of log10‐transformed FPKM values across all samples. (b) Principal component analysis (PCA) revealed clear clustering of samples according to developmental stage and nutritional condition.

### 3.3 Longitudinal Analysis of DEGs and Nutrient Transition Enrichment in the Feeding Group

The transition from endogenous yolk dependence to stable exogenous feeding in *L. longirostris* involves coordinated transcriptomic changes. The number of DEGs increased from 211 (F3 vs. F2) to 1058 during the transition to the mixed nutrition phase (F5 vs. F3), and this increase intensified during the transition to full exogenous feeding (F9 vs. F5; 1354 DEGs) and in the stabilization phase (F14 vs. F9; 1368 DEGs) (Supplementary Table 3). This progression reflects the extensive molecular adaptations necessary for the shift from dependence on the yolk sac to active predation.

Despite these changes, several core functional categories remained enriched across stages. GO analysis revealed persistent enrichment of “cellular anatomical entity,” “binding,” and “catalytic activity,” suggesting a continued need for protein synthesis and catalytic processes during organogenesis. In parallel, pathways related to amino acid biosynthesis, carbohydrate metabolism, and lipid sensing remained active throughout development (Supplementary Figure 1). Supplementary Figure 1 presents longitudinal functional enrichment analysis of DEGs during nutritional transition.

Beyond these shared features, each developmental transition was characterized by distinct physiological mechanisms, reflecting key milestones in larval development. In F3 vs. F2, DEGs were primarily associated with cell adhesion molecules and neuroactive ligand‐receptor interaction, implying early preparation of neural and muscular systems. Before the onset of exogenous feeding, glycolysis and amino acid biosynthesis were activated in the larvae to support the development of sensory and motor systems, providing a structural basis for successful initial feeding. In F5 vs. F3, ribosome and oxidative phosphorylation pathways showed strong enrichment, indicating enhanced translational activity and aerobic metabolism (Horvath et al. 2024; Wang et al. 2022). Glutathione metabolism was activated as an important antioxidant defense to scavenge free radicals and alleviate metabolic damage (Giustarini et al. 2023). The activation of the cardiac muscle contraction pathway reflects the physiological demand for high circulatory efficiency to support active predation (Xu et al. 2023). Together, increased protein synthesis, aerobic metabolism, circulation, and antioxidant capacity enable larvae to sustain feeding and handle metabolic byproducts. In F9 vs. F5, when larvae reached the fully exogenous feeding stage, the focus shifted toward systemic integration and establishment of the barrier. The enrichment of extracellular matrix (ECM)‐receptor interaction and phagosome pathways suggests maturation of tissue scaffolding (Ye et al. 2024). This stage marked a developmental transition: the activation of the intestinal immune network for the synthesis of immunoglobulin A (IgA). This suggests that the gastrointestinal tract had evolved from a simple absorptive organ into a sophisticated immunological barrier, capable of regulating interactions between the host and the environment (Valenzuela et al. 2021). As the adaptive immune system confers specific, memory‐based pathogen defense, rapid immune maturation is important for fry to resist infection while adapting to exogenous diets (Nishiguchi et al. 2024). The peroxisome proliferator‐activated receptor (PPAR) signaling pathway and the biosynthesis of unsaturated fatty acids are significantly enriched. Key lipid genes (*PPARα*, *PPARβ*, *SCD1*) in the PPAR pathway modulate lipid metabolism and promote lipid synthesis, supporting stable lipid homeostasis under exogenous feeding (Zheng et al. 2024). ECM and intestinal immune maturation remodel the gut into a defensive barrier; coupled with activated lipid metabolism, this allows larvae to utilize dietary lipids efficiently and sustain homeostasis in microbe‐rich environments. In F14 vs. F9, the stabilization stage was characterized by physiological refinement, with steroid biosynthesis and unsaturated fatty acid biosynthesis dominating molecular changes. Changes in lipid profiles, especially long‐chain polyunsaturated fatty acids, enhance membrane fluidity and metabolic efficiency (Redouane et al. 2023). Suppression of DNA replication and regulation of ferroptosis indicate a shift from rapid growth to improved nutrient utilization and maintenance of systemic homeostasis.

The nutritional transition in *L. longirostris* is a systematic developmental process governed by distinct molecular milestones. By integrating Venn diagram analysis with PPI networks, we identified the core regulators that persist throughout the transition and the stage‐specific hubs associated with developmental progression.

By conducting longitudinal analysis, we identified 15 core DEGs (F2 through F14), which represented the fundamental molecular framework of larval ontogeny. The results of the KEGG enrichment analysis revealed that these common DEGs were primarily associated with lipid metabolism (unsaturated fatty acid biosynthesis and fatty acid elongation) and cell proliferation signaling (mTOR and MAPK pathways) (Figure 3). This coordinated network provides the carbon skeleton and energy precursors required for membrane biogenesis, lipid signaling, and developmental remodeling. Within this framework, elongation of very long chain fatty acids protein 2 (*elovl2*) and chitinase, acidic.2 (*chia.2*) were identified as key hubs. The persistent activation of *elovl2* suggests a continued requirement for long‐chain polyunsaturated fatty acid synthesis to support neural and structural development (Wang et al. 2023), while *chia.2* probably facilitates metabolic adaptation. These results highlight a conserved, lipid‐centered metabolic program as the cornerstone of physiological maturation in *L. longirostris* .

**FIGURE 3:**
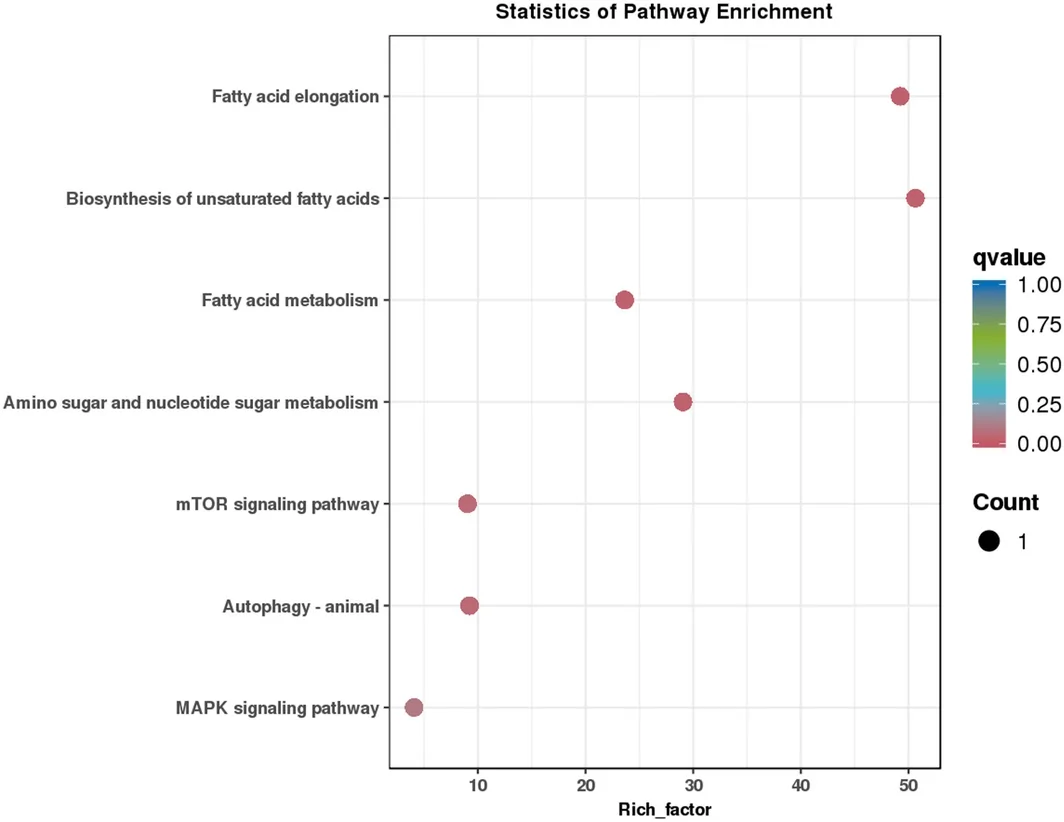
KEGG enrichment analysis of common DEGs across all nutritional transition stages.

At the onset of exogenous feeding (F5), the larval transcriptome expanded markedly. We identified 404 “opening‐specific” genes that define this initiation phase (Figure 4a). The Venn diagram illustrates the filtering strategy used to identify 404 stage‐specific genes. The transcriptomic profile shifted toward rapid growth and energy mobilization. According to KEGG enrichment analysis (Figure 4b), pathways related to the ribosome and oxidative phosphorylation were significantly upregulated. To further identify key regulators at this stage, we screened 76 DEGs from the four most significant pathways (*q* < 0.05). PPI analysis identified key hubs, including *rpl29* and *rpl35* (ribosomal proteins), along with *atp5mf, ndufab1b*, and *ndufa1* (NADH dehydrogenase subunits) (Figure 5a). The upregulation of these genes suggests a rapid mobilization of cellular resources to increase protein synthesis and ATP production, supplying energy for active feeding. Additionally, the early activation of glutathione metabolism may protect against oxidative stress during this metabolic shift.

**FIGURE 4:**
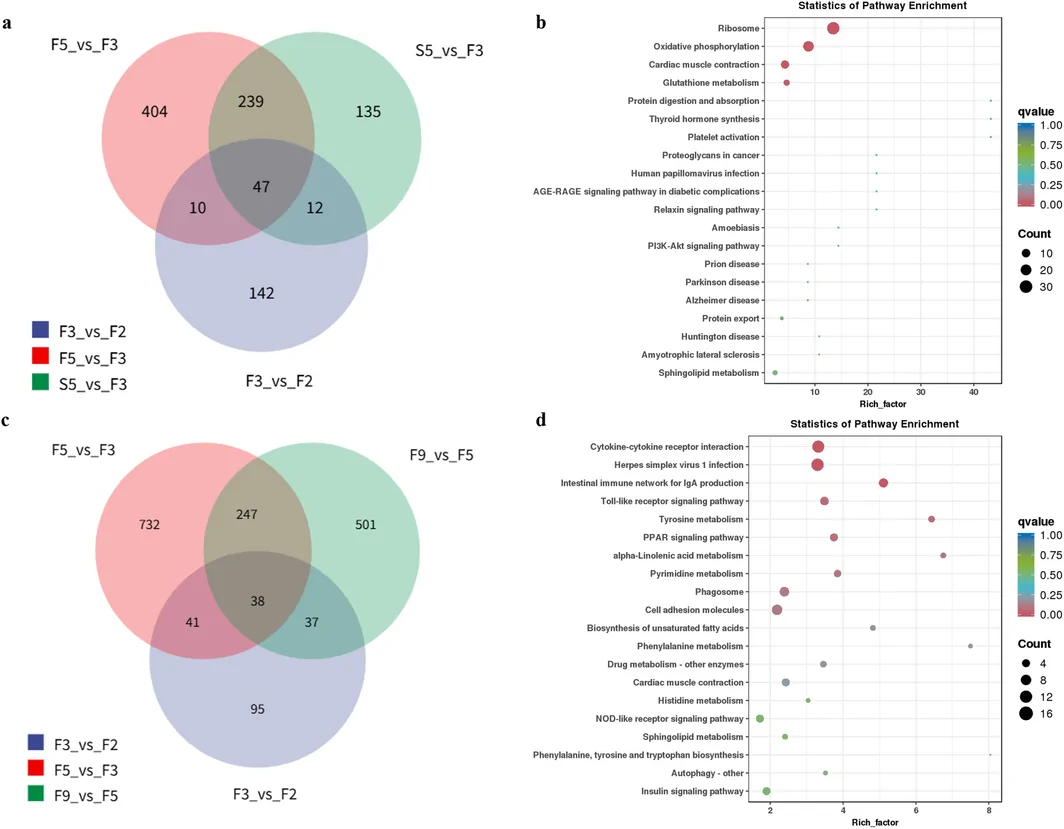
(a) The Venn diagram shows 404 genes specifically regulated during the transition to the mixed nutrition phase (F5 stage). Filtering criteria: Significant upregulation in F5 vs. F3, coupled with nonsignificant change or downregulation in both S5 vs. F3 and F3 vs. F2. (b) KEGG enrichment analysis of the 404 stage‐specific genes identified in the F5 transition. (c) The Venn diagram shows stage‐specific DEGs for the transition to full exogenous nutrition (F9 stage). Filtering criteria: Upregulation in F9 vs. F5, with nonsignificant differences or downregulation in both F5 vs. F3 and F3 vs. F2. (d) KEGG enrichment analysis of the identified F9‐specific genes.

**FIGURE 5:**
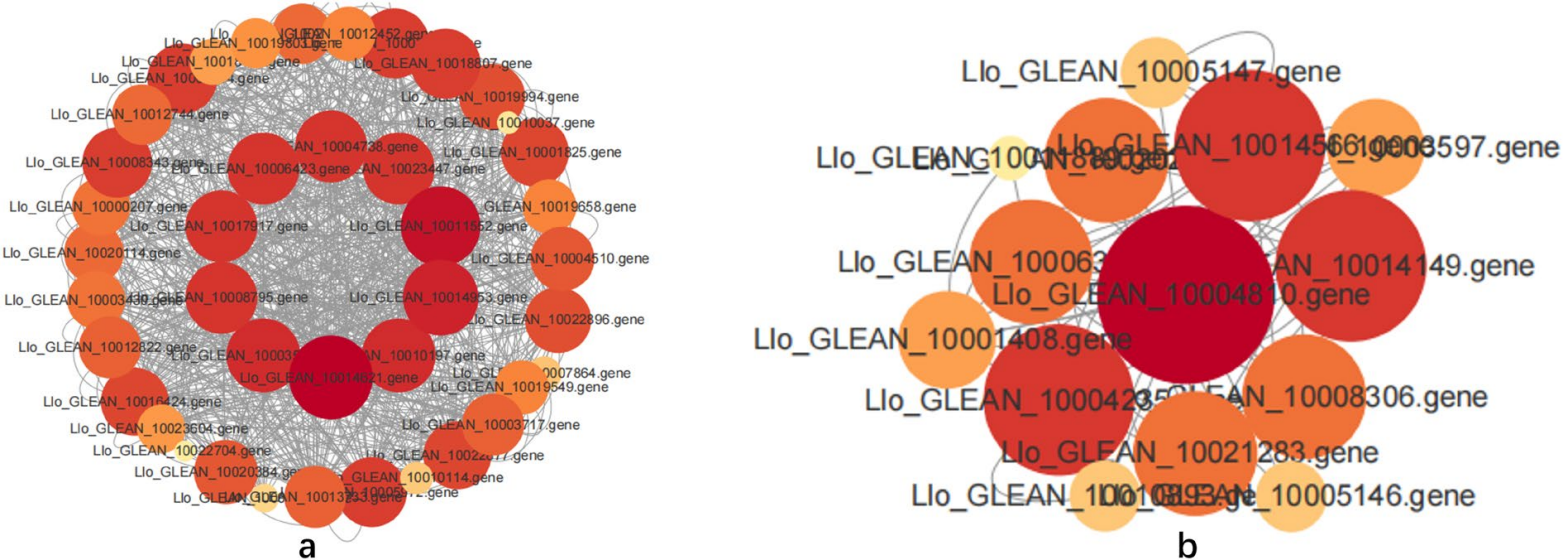
PPI networks of stage‐specific genes during the nutritional transition of *L. longirostris* larvae. (a) PPI network of the F5 stage. (b) PPI network of the F9 stage.

The transition to full exogenous nutrition (F9) was characterized by changes in 501 stage‐specific genes (Figure 4c). The Venn diagram illustrates the filtering strategy used to identify these genes, which were predominantly enriched in immune‐related and infection‐related pathways (Figure 4d). These genes were involved in herpes simplex virus 1 infection (associated with host antiviral defense), intestinal immune network for the production of IgA, and the Toll‐like and NOD‐like receptor signaling pathways, which are major components of the innate immune system (Li et al. 2022). Significant enrichment was also observed in cytokine‐cytokine receptor interaction, a key process underlying immune cell communication and inflammatory responses. To identify the regulatory hubs involved in this immune maturation, we screened 31 DEGs from the three most significant pathways (*q* < 0.05). PPI analysis identified key hubs, including *socs3a, cxcr4b*, and *nfkbiaa* (Figure 5b). These findings indicated that F9 is a critical window for establishing the intestinal mucosal barrier and fine‐tuning inflammatory regulation, enabling larvae to adapt to environmental pathogens during the transition to fully exogenous feeding.

The nutritional transition of *L. longirostris* is characterized by a three‐phase molecular program: (1) an initial increase in ribosomal and energetic pathways during the mixed nutrition phase; (2) the integration of PPAR‐mediated metabolism and intestinal immune networks for structural maturation; (3) a shift toward redox regulation and homeostatic maintenance. This coordinated sequence promotes the transition from endogenous maternal reserves to environmental self‐sufficiency.

### 3.4 Analysis of DEGs and Enrichment Analysis of Stress Progression in the Starvation Group

The transcriptomic response of *L. longirostris* larvae to progressive starvation is characterized by a hierarchical intensification of molecular disarray, reflecting attempts to maintain physiological function under increasing energy deficiency. The number of DEGs increased markedly, rising from 685 (comparing S5 with F3) to 7087 (comparing S14 with S9), suggesting widespread changes in transcriptional regulation with the depletion of nutritional reserves (Supplementary Table 4).

A persistent re‐tuning of cellular metabolic processes and biological regulation occurred across all starvation phases (Supplementary Figure 2). Supplementary Figure 2 shows GO and KEGG enrichment analysis of DEGs in the starvation group across critical developmental transitions. This universal signature manifested as the continuous activation of nutrient‐sensing hubs, especially the PPAR signaling pathway. Throughout starvation, the larval transcriptome emphasized mobilization of endogenous macromolecules, shifting from a state of local compensatory adaptation to a widespread, nonspecific catabolism. This common trajectory highlights the progression from adaptive survival to metabolic collapse as larvae approach the PNR stage.

Under starvation, *L. longirostris* larvae exhibit a staged physiological deterioration. At S5, larvae exhibit prioritization of vital organs: PPAR‐mediated lipolysis helps maintain circulation and tissue structure while limiting protein synthesis (Gao et al. 2023). In addition, regulation of cardiac muscle contraction and cell adhesion molecules (CAMs) implies prioritization of circulatory and structural integrity, which depends on energy from fatty acid and glucose oxidation; this resource allocation helps mitigate the effects of food deprivation (Yang et al. 2024). This compensatory state shifts to energy‐conserving survival mode by S9, with coordinated repression of spliceosome and cell‐cycle pathways to minimize ATP consumption, yet this protection is transient. By Day 14, when the PNR was reached, starved larvae displayed irreversible physiological impairments. Widespread catabolism and increased proteasome activity promote protein breakdown to meet basal energy requirements. Disruption of ECM–receptor interactions and focal adhesions contributes to severe tissue damage, impairing the intestinal barrier and reducing the capacity for nutrient uptake even upon refeeding. Depressed MAPK signaling marks the collapse of pro‐survival networks. Overall, starving larvae prioritize core functions over growth to prolong survival. Ultimately, tissue breakdown impairs their ability to swim and feed, leading to irreversible damage that prevents recovery even if food becomes available.

#### 3.4.1 KEGG Pathway Enrichment of Common DEGs in the Starvation Group

Across the three stages of progressive starvation, 140 common DEGs were identified as core genes involved in the starvation stress response (Figure 6a). The Venn diagram illustrates the filtering strategy. An enrichment analysis was performed based on the KEGG database (Figure 6b). DEGs were enriched in ferroptosis, glutathione metabolism, and mTOR signaling, indicating oxidative injury, glutathione‐dependent antioxidant response, and mTOR‐mediated metabolic suppression as key molecular signatures of starvation stress.

**FIGURE 6:**
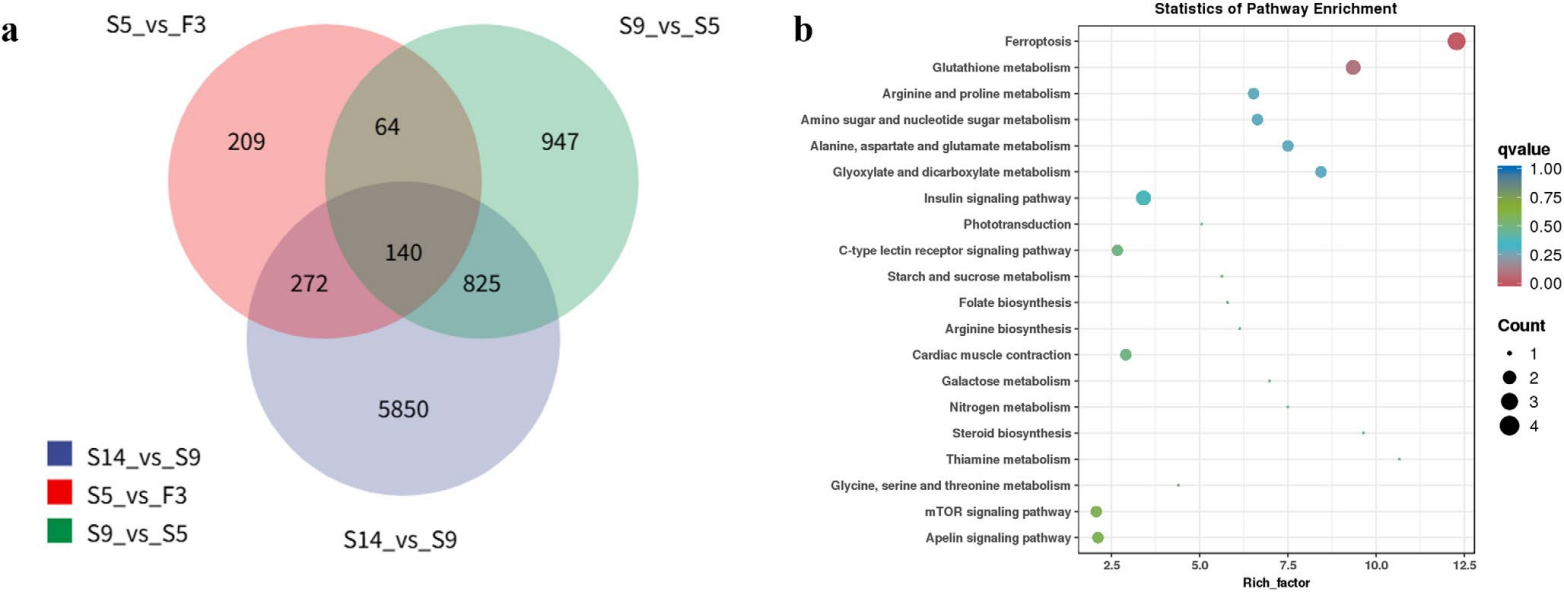
(a) The Venn diagram shows common DEGs across progressive starvation stages (S5 vs. F3, S9 vs. S5, S14 vs. S9). (b) KEGG pathway enrichment analysis of the 140 core starvation‐responsive genes.

#### 3.4.2 WGCNA Analysis and PNR Stage Key Gene Screening

To identify the gene regulatory networks that regulate the transition to the PNR stage, WGCNA was performed. Correlation analysis between modules and starvation stages identified a turquoise module (comprising 2414 genes) that demonstrated a positive correlation with the S14 stage (*r* = 1, *p* = 2 × 10^−4^), but was negatively correlated with the early endogenous (F3) and mid‐starvation (S9) stages (Figure 7a). This stage‐specific expression pattern suggests that the turquoise module captures molecular signatures associated with the PNR. Overlap analysis of this module and the 5850 DEGs identified during the S14–S9 transition identified a core set of 1679 genes, primarily enriched in two functional clusters: immune regulation and programmed cell death. Figure 6b displays KEGG enrichment analysis of the 1679 core genes (Figure 7b).

**FIGURE 7:**
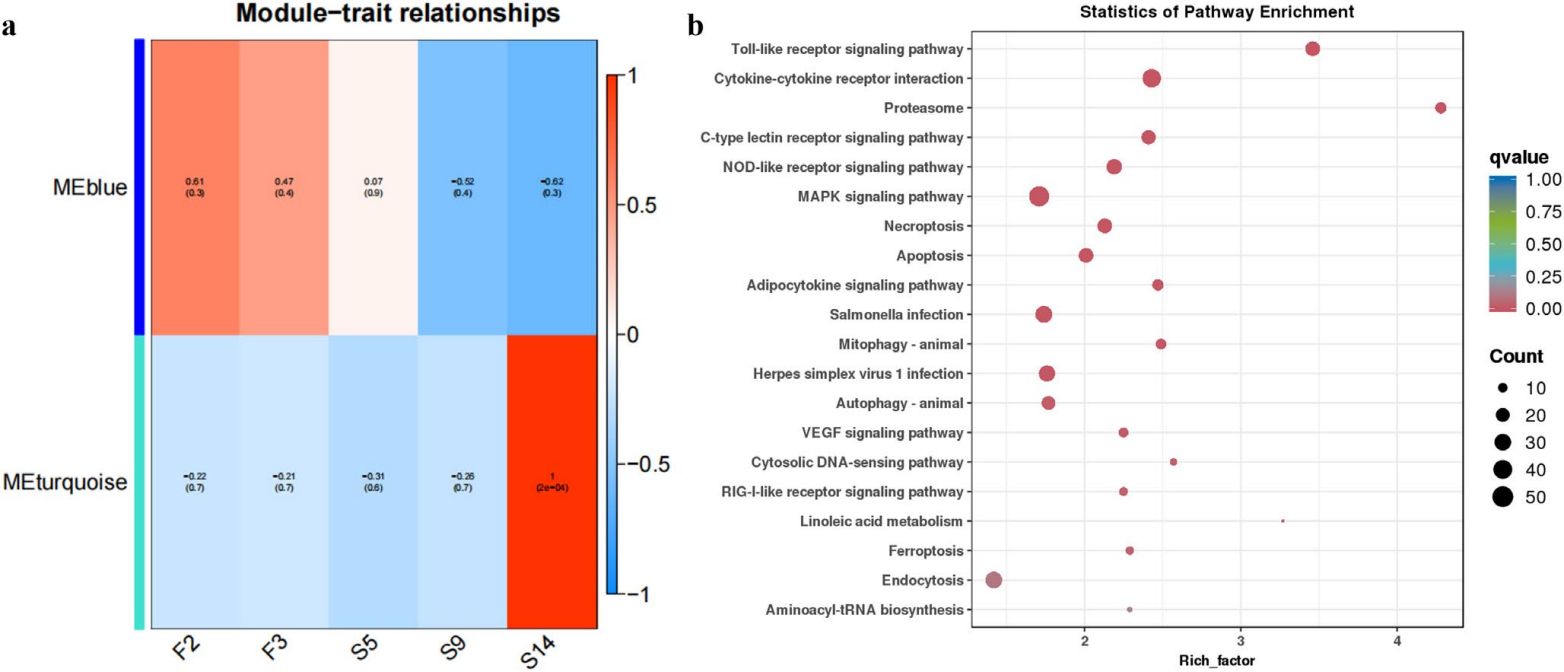
Identification and functional characterization of the gene regulatory network driving the PNR stage. (a) The heatmap shows the module‐trait relationship based on WGCNA. (b) KEGG enrichment analysis of PNR‐associated core genes. The bubble plot shows significantly enriched pathways of 1679 core genes (identified as the intersection between the METurquoise module and S14 vs. S9 DEGs).

The terminal stage of starvation in *L. longirostris* is characterized by a widespread activation of innate immune pathways. Toll‐like receptor (TLR), NOD‐like receptor, and C‐type lectin receptor pathways were significantly enriched. Larvae may recognize and respond to damage‐associated molecular patterns released from degrading tissues. This immune activation coincides with multiple cell death programs: autophagy, mitophagy, apoptosis, ferroptosis, and necroptosis. Autophagy and mitophagy may support compensatory recycling, whereas concurrent ferroptosis and necroptosis suggest a shift toward proinflammatory lytic cell death.

The intersection between pathway genes with *q* < 0.05 and turquoise module genes was determined. WGCNA was conducted on 174 genes with expression levels > 0. The top 15 core regulatory genes were selected through PPI analysis (Supplementary Table 5) (Figure 8a). Supplementary Table 5 lists the hub genes identified from the turquoise module PPI network. The table lists gene IDs and functional families. The central network arrangement highlights a cluster of 15 core regulatory genes with the highest degree of connectivity. The connections between these hubs and peripheral nodes suggest that PNR is governed by a highly integrated transcriptional program rather than isolated changes in gene expression. Hierarchical clustering of the 15 candidate genes showed a distinct expression peak at the PNR. This stage‐specific upregulation indicates that these genes may be strongly associated with the terminal response to starvation and the irreversible transition into the PNR state (Figure 8b).

**FIGURE 8:**
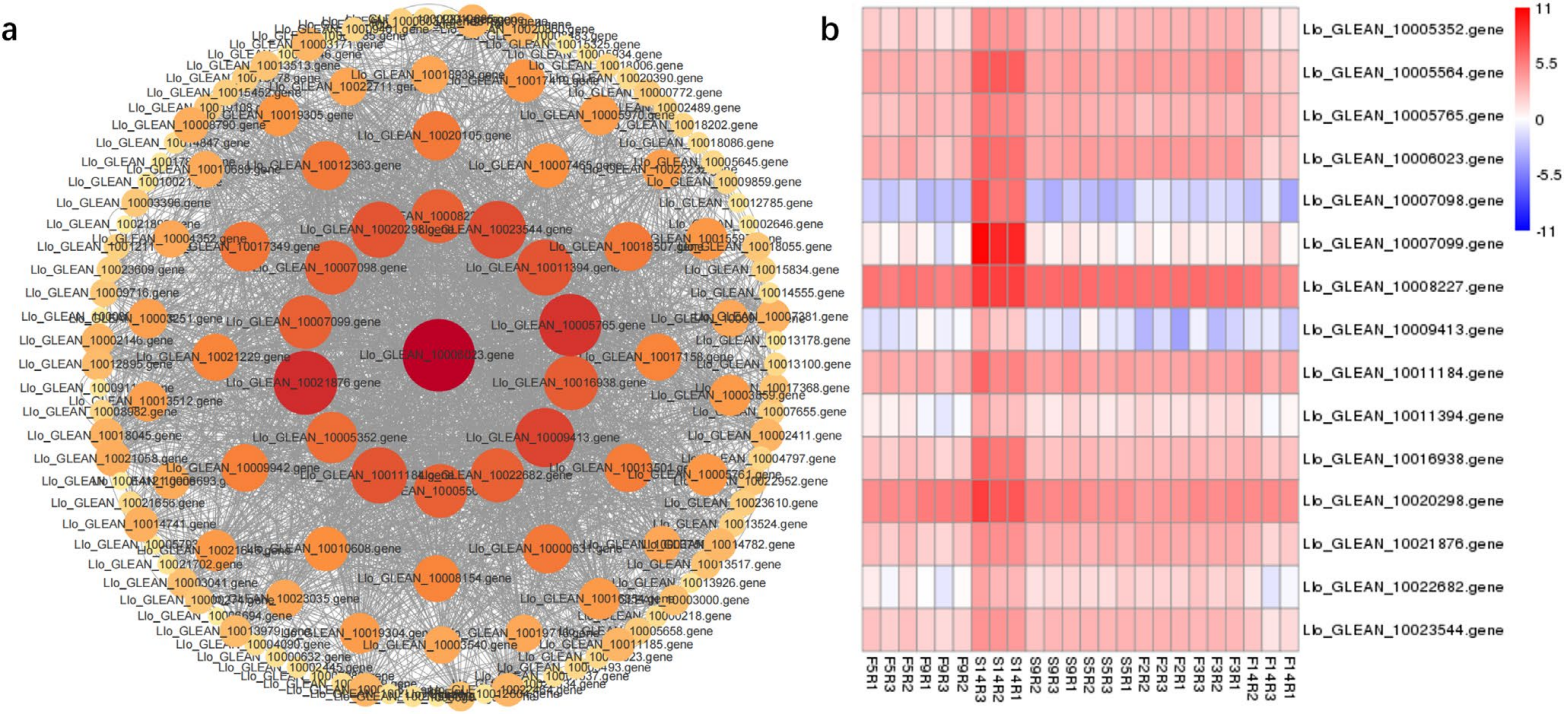
(a) The PPI network of core regulatory genes at the PNR stage is illustrated. (b) Hierarchical clustering analysis of 15 selected genes across developmental stages.

Based on the 15 core regulatory genes identified by WGCNA, the PNR transcriptome was strongly associated with TNF receptor‐mediated necroptosis. ATP depletion may impair cIAP1 activity, disrupt RIPK1 ubiquitination, and shift TNFR1 signaling from survival toward RIPK1–RIPK3–MLKL‐mediated cell death (Ishaq et al. 2025; Martínez‐Osorio et al. 2026). This ATP‐dependent switch likely underlies the irreversible nature of PNR in *L. longirostris* larvae. Consistent with this, KEGG enrichment indicated activation of innate immune pathways, including NOD‐like receptor signaling and cytokine–cytokine receptor interaction, suggesting that necroptotic cell death may amplify inflammatory signaling through DAMP release (Yao et al. 2025). In addition, *Llo_GLEAN_10022682*, annotated as a phosphatidylinositol 3‐kinase catalytic subunit, was correlated with the PNR stage and enriched in pathways related to PI3K‐Akt, mTOR, and focal adhesion, suggesting a possible role in energy sensing and stress adaptation during starvation‐induced collapse (Yuan et al. 2013).

In parallel, sustained activation of the NF‐κB pathway appears to contribute to an inflammatory lock at the PNR stage. Genes associated with NF‐κB signaling, including *Llo_GLEAN_10021876* and *nfkbiaa*, suggest sustained inflammatory activation. During prolonged energy deficiency, the resynthesis of IκBα may be compromised, thereby delaying termination of NF‐κB signaling and maintaining p65 activity in the nucleus. This may promote sustained expression of proinflammatory cytokines, such as IL‐1β, while suppressing anabolic recovery processes required for successful refeeding (Sun and Jiang 2026). The concomitant downregulation of ECM–receptor interaction and focal adhesion pathways further suggests progressive loss of tissue integrity, which may limit the possibility of recovery once PNR is reached.

STAT3 and STAT6 may further sustain this inflammatory state by promoting cytokine‐driven feedback amplification downstream of persistent NF‐κB activation. In parallel, reduced *traf3* expression and impaired IκBα resynthesis may weaken negative feedback control, resulting in prolonged nuclear retention of p65 and sustained inflammatory transcription at the PNR stage (Yu et al. 2009). Together, these changes suggest that PNR is characterized by a self‐sustaining inflammatory and necroptotic state rather than merely a passive response to starvation. Moreover, low intracellular ATP levels may promote AMPK‐dependent phosphorylation and inactivation of Bcl‐xL, thereby favoring Bax/Bak activation and mitochondrial outer membrane permeabilization (Huang et al. 2019); collectively, these ATP‐dependent alterations converge on RIPK3–MLKL‐mediated necroptotic membrane rupture, consistent with the irreversible death depicted in Figure 9 (Wang et al. 2014). These transcriptomic signatures may therefore serve as candidate indicators for identifying an ecological threshold at which larval survival becomes irreversible.

**FIGURE 9:**
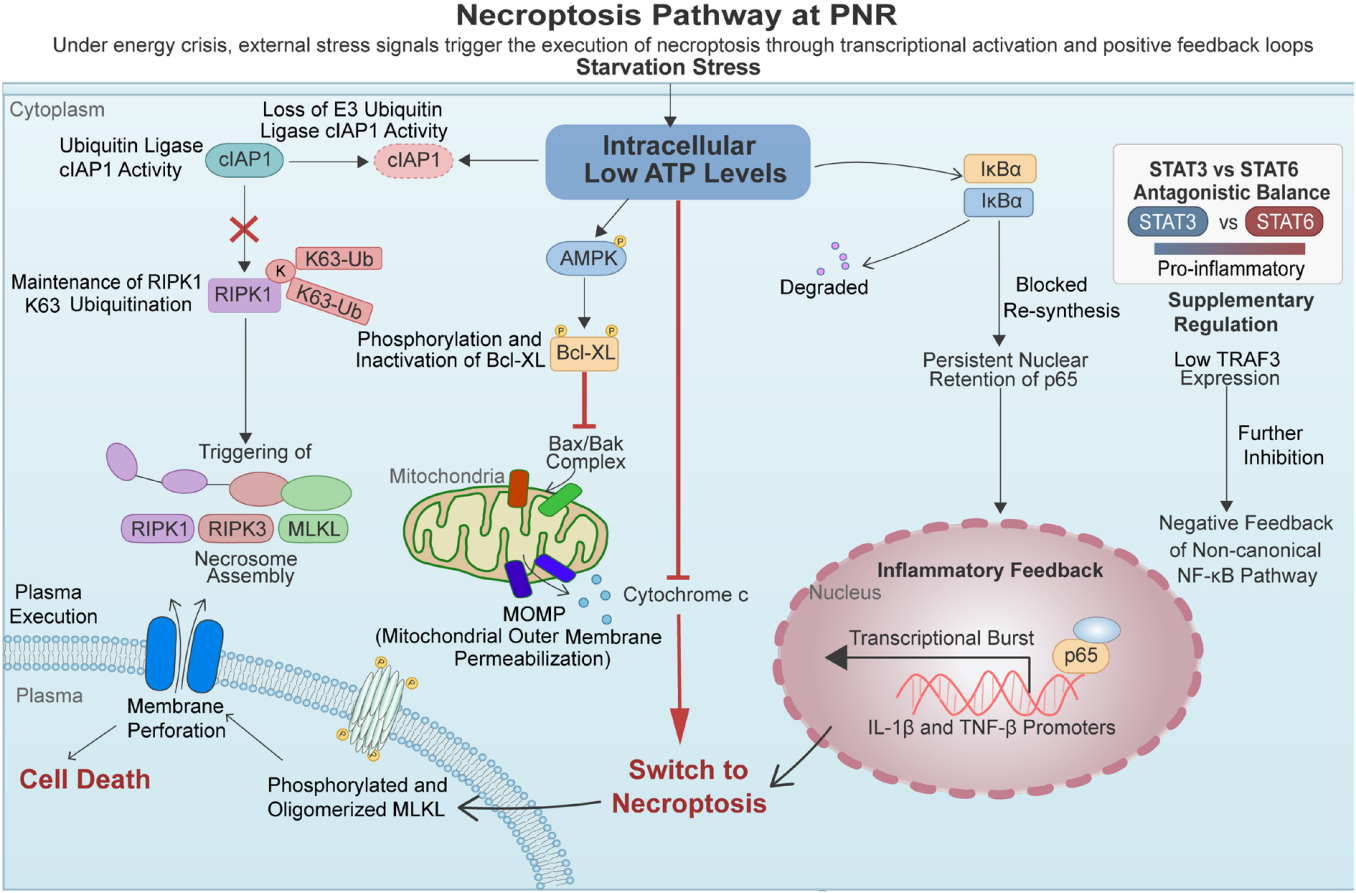
The necroptosis pathway associated with the PNR stage is illustrated.

### 3.5 Interference Effects of the Feeding Group and the Starvation Group in the Same Period

By integrating longitudinal changes in the feeding group (F‐groups) with patterns of response to starvation (S‐groups), we examined how nutritional deprivation progressively alters the developmental trajectory of *L. longirostris* larvae. The number of DEGs increased markedly, rising from 240 during the mixed nutrition phase (S5 vs. F5) to 8897 at the PNR stage (S14 vs. F14) (Supplementary Table 6). This marked increase in transcriptomic divergence suggests a shift from the early‐phase metabolic compensation to the late‐phase systemic collapse.

We identified 93 consensus DEGs across all starvation stages as the molecular baseline of starvation stress. These genes were mainly enriched in oxidative phosphorylation, fatty acid metabolism, the insulin signaling pathway, and the mitogen‐activated protein kinase (MAPK) signaling pathway. In the feeding group, the combined actions of the insulin and phosphatidylinositol 3‐kinase (PI3K) pathways maintained anabolic activity and supported rapid growth. Conversely, starvation induced a pronounced anabolic–catabolic imbalance, in which a shift toward excessive catabolism compromised the metabolic infrastructure essential for homeostatic stability even prior to the manifestation of physical symptoms.

During the transition from endogenous nutrition to mixed nutrition, starvation induces rapid metabolic reprogramming. MAPK pathway activation contributes to the stress response. The enrichment of the insulin and PPAR signaling pathways implies that larvae maintain glucose homeostasis by mobilizing lipid reserves. The downregulation of protein processing in the endoplasmic reticulum and tryptophan metabolism indicates that energy‐intensive processes, such as protein translation, are restricted early to conserve ATP (Supplementary Figure 3a,b). Supplementary Figure 3 presents stage‐specific KEGG pathway enrichment analysis for cross‐sectional comparisons.

During the middle stage of nutrient deprivation (Day 9), transcriptomic remodeling indicates increased physiological responses to worsening nutritional stress. Enriched “response to stimulus” and “ATP binding”, together with changes in ribosomal structural components and oxidoreductase activity, indicate a bioenergetic shift: larvae limit energy‐demanding translation and enhance oxidoreductase‐related pathways as an adaptive response to prolonged food deprivation (Supplementary Figure 3c,d).

At the PNR (S14 vs. F14), KEGG enrichment was primarily associated with oxidative phosphorylation, ribosome, cardiac muscle contraction, and ECM–receptor interaction. This was consistent with the GO CC category, which indicated extensive disruption in cellular anatomical entities and protein complexes. Oxidative phosphorylation is enriched, signaling a bioenergetic crisis. Larvae consume muscle and other tissues to sustain basal functions. This process causes systemic degradation, leading to organ failure (Supplementary Figure 3e,f).

Starvation forces larvae to trade growth for survival by mobilizing internal reserves. Although this permits short‐term survival, the resulting degradation of muscle and tissue eventually destroys the physical capacity required for foraging. By the PNR stage, this tissue consumption reaches a critical level, making it impossible for larvae to resume normal development even if food is restored. These results agree with the longitudinal trends observed in the starvation group, reinforcing the conclusion that prolonged nutritional deprivation leads to an irreversible loss of physiological fitness.

### 3.6 Real‐Time Quantitative PCR Verification

Real‐time quantitative PCR analysis was performed for seven genes, including *rpl35, ndufa1, socs3a, nfkbiaa, stat3, traf3*, and *Llo_GLEAN_10021876*. The qPCR results agreed with the expression trends observed in the RNA‐seq data, which indicated that the transcriptome sequencing data were reliable and the screening of DEGs was accurate (Figure 10).

**FIGURE 10:**
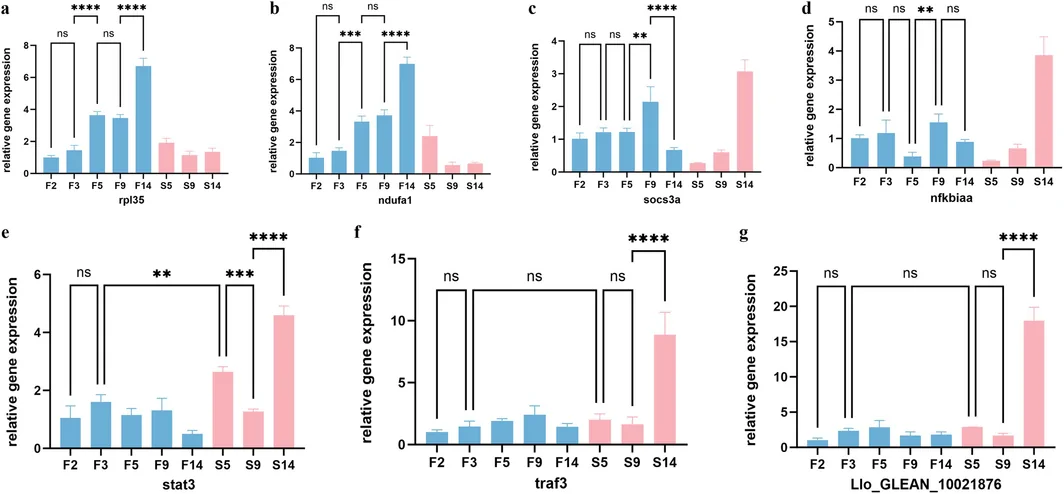
Real‐time quantitative PCR verification was performed for seven genes. (a) *rpl35*, (b) *ndufa1*, (c) *socs3a*, (d) *nfkbiaa*, (e) *stat3*, (f) *traf3*, (g) *Llo_GLEAN_10021876*.

## 4 Discussion

This study presents a transcriptomic framework for the nutritional transition and starvation‐induced PNR in *L. longirostris* larvae. Under normal feeding conditions, the transition from endogenous yolk dependence to exogenous nutrition was characterized by a coordinated developmental program involving translational activation, bioenergetic expansion, immune‐barrier maturation, and improved nutrient utilization. Under starvation, larvae displayed a hierarchical trajectory from early compensatory metabolic reorganization to terminal systemic collapse. At the PNR stage, the transcriptomic landscape shifted toward proteolytic catabolism, loss of ECM–receptor and focal‐adhesion integrity, and activation of programmed cell death and inflammatory signaling. WGCNA further identified a PNR‐associated module enriched in necroptosis and inflammatory pathways, supporting a mechanistic model linking bioenergetic failure to irreversible larval death.

Normal nutritional transition in *L. longirostris* progresses from biomass accumulation to functional maturation. At first feeding, enriched ribosomal and oxidative phosphorylation pathways support translation and aerobic energy supply needed for active feeding and growth (Horvath et al. 2024; Wang et al. 2022). Subsequent activation of ECM–receptor interaction, intestinal immune networks and lipid metabolism supports remodeling of the gut from an absorptive surface into an immune and metabolic barrier (Valenzuela et al. 2021; Zheng et al. 2024). Changes in lipid profiles and reduced DNA replication later shift the program from rapid growth to homeostasis and membrane optimization (Redouane et al. 2023; Wang et al. 2023). Successful transition therefore requires coordinated maturation of protein synthesis, energy metabolism, immunity and tissue structure.

Early starvation redirects limited energy to core functions: PPAR‐driven lipid mobilization accompanies suppressed protein synthesis and cell cycling to reduce anabolic energy demands (Gao et al. 2023; Yang et al. 2024). This compensatory response is temporary. Prolonged starvation promotes ATP conservation and catabolism; at the PNR, proteasome activation and disrupted ECM–receptor and focal adhesion signaling occur. As these structures support cell survival, tissue organization and barrier function, their disruption impairs intestinal and hepatic nutrient‐processing capacity, limiting recovery after refeeding.

The PNR module supports a model in which energy deficiency triggers self‐amplifying necroptosis and inflammation. Low ATP impairs RIPK1 ubiquitination, switching TNFR1 signaling to RIPK1–RIPK3–MLKL‐dependent necroptosis (Ishaq et al. 2025; Martínez‐Osorio et al. 2026). Impaired IκBα turnover sustains NF‐κB activity, while altered TRAF3 and STAT3/STAT6 signaling weaken negative feedback on inflammation (Sun and Jiang 2026; Yu et al. 2009). ATP deficiency also promotes AMPK‐mediated Bcl‐xL suppression and mitochondrial permeabilization, aggravating membrane rupture (Huang et al. 2019; Wang et al. 2014). These signatures indicate an active inflammatory–necroptotic cascade, rather than simple energy exhaustion. This model provides a mechanistic explanation for why refeeding after the PNR fails to restore normal development.

A comparative perspective across teleosts indicates that the transition from endogenous to exogenous nutrition, as well as the occurrence of the PNR, is governed by both conserved and species‐specific regulatory programs. In *Seriola rivoliana* , the onset of feeding is accompanied by marked transcriptomic reprogramming in energy metabolism and digestive maturation (Guerrero‐Tortolero et al. 2021), whereas delayed first feeding in *carp* significantly impairs growth and survival (Feng et al. 2025). After 14 days of starvation, the larvae of *Micropterus salmoides* exhibited significant decreases in serum glucose, cholesterol, triglyceride, and alkaline phosphatase concentrations, accompanied by changes in the intestinal microbiota and transcriptome (Wang et al. 2024), highlighting the narrow developmental window for successful nutritional transition. These patterns are consistent with the present results in *L. longirostris* , in which larvae showed a progressive shift from reserve mobilization to systemic failure under starvation. However, interspecific differences may reflect variation in life‐history strategy, developmental pace, and ecological niche. Compared with species such as *S. rivoliana* and carp, benthic catfish‐like taxa are generally thought to depend more strongly on endogenous reserves and sensory‐mediated foraging during early development, which may contribute to the stage‐dependent transcriptomic changes observed near the PNR in *L. longirostris* . Together, these comparisons suggest that the nutritional transition in *L. longirostris* is built on a conserved teleost framework but shaped by species‐specific ecological adaptations. In this context, the distinct transcriptional signatures identified in our study may reflect the molecular effects of these adaptations, defining the boundaries of larval life status and survival potential.

These findings have direct practical implications for aquaculture. Within a potential molecular diagnostic framework, the ribosomal burst at F5 may indicate a critical first‐feeding window requiring protein‐rich diets, whereas the NF‐κB/STAT3 axis may serve as a candidate early‐warning indicator of larval health before irreversible damage occurs. Collectively, these markers provide a preliminary framework for evaluating larval viability and recruitment risk, and may help assess recruitment potential in wild Siluriformes populations. However, this study has several limitations. First, the proposed necroptosis–inflammatory model is inferred from transcriptomic signatures and pathway enrichment; direct evidence from protein phosphorylation, pathway inhibition, histopathology, or cellular functional assays is needed to establish causality. Second, the absence of complementary physiological metrics (e.g., digestive enzymes and oxidative stress) and environmental covariates (e.g., temperature and dissolved oxygen) limits our biological interpretation and may confound nutritional stress responses. Therefore, further experiments are needed to verify the causal roles of necroptosis–inflammation in PNR and to improve biological interpretation. Researchers should focus on the following crucial directions: (i) combining phosphoproteomics, targeted pathway inhibition, histopathology, and cellular assays to test the necroptosis–inflammatory model; (ii) integrating digestive enzyme, oxidative stress, and energy metabolite measurements with transcriptomic profiling; and (iii) monitoring or manipulating temperature and dissolved oxygen under different rearing conditions to establish a multidimensional framework for larval quality assessment.

## 5 Conclusions

This study establishes a high‐resolution transcriptomic framework for the nutritional transition and starvation‐induced PNR in *L. longirostris* . The findings describe a three‐phase molecular program in normal ontogeny: translational activation (F5), immune‐barrier maturation (F9), and enhanced nutrient utilization (F14). Under starvation, a progressive transcriptomic trajectory was identified, shifting from early adaptive metabolic realignment and translational arrest to terminal mitochondrial failure and widespread tissue proteolysis. Based on these molecular signatures, a mechanistic model for the PNR stage is proposed, which is characterized by a transition from compensatory autophagy to necroptotic execution and inflammatory locking through the NF‐κB/STAT3 axis. This cumulative molecular injury may explain why refeeding after the PNR fails. These results advance PNR from a phenomenological observation to a mechanistic model with ecological relevance. The identified pathways and genes therefore offer candidate biomarkers for improving larval rearing and for predicting feeding success, survival potential, and recruitment risk in wild Siluriformes populations.

## Author Contributions

**Shanshan Guan:** data curation (equal), formal analysis (equal), methodology (equal), validation (equal), visualization (equal), writing – original draft (equal). **Xiangchao Si:** investigation (equal), visualization (equal). **Jinhui Yu:** methodology (equal), validation (equal), visualization (equal). **Jianfang Guo:** methodology (equal), validation (equal). **Ruixuan Qi:** methodology (equal), visualization (equal). **Meng Yuan:** methodology (equal), validation (equal). **Yongtao Tang:** methodology (equal), project administration (equal), supervision (equal). **Qiang Li:** investigation (equal), resources (equal). **Chuanjiang Zhou:** conceptualization (equal), funding acquisition (equal), methodology (equal), project administration (equal), supervision (equal), writing – review and editing (equal).

## Funding

This work was supported by the following funding bodies: Sichuan Freshwater Fish Innovation Team of the National Modern Agricultural Industrial Technology System, “1 + 9” open competition mechanism to select the best candidates and scientific and technological project of Sichuan Academy of Agricultural Sciences (1 + 9KJGG004), the Henan Province Department of Science and Technology (182102110046, 222102110294), and the Training Plan of Young Excellent Teachers in Colleges and Universities of Henan Province (2019GGJS063).

## Ethics Statement

The animal study protocol was reviewed and approved by the Ethics Committee of the Fisheries Research Institute, Sichuan Academy of Agricultural Sciences (No. 20240423001A, approved on 23 April 2024), and was conducted in accordance with the relevant national Guidelines for the Care and Use of Laboratory Animals.

## Conflicts of Interest

The authors declare no conflicts of interest.

## Supporting information


**Supplementary Table 1:** Primer sequences used for qRT‐PCR validation in *Leiocassis longirostris*.
**Supplementary Table 2:** Summary of RNA sequencing quality control metrics for all samples.
**Supplementary Table 3:** Number of DEGs at each stage of nutritional transition in the normal group.
**Supplementary Table 4:** Number of DEGs at each stage of stress escalation in the starvation group.
**Supplementary Table 5:** The top 15 core regulatory genes.
**Supplementary Table 6:** Statistical comparison of the quantity of DEGs between the normal groups and the starvation groups within the same period.
**Supplementary Figure 1:** Longitudinal functional enrichment analysis of DEGs during nutritional transition. (a) GO enrichment bar plot of DEGs in the F3 vs. F2 comparison (pure endogenous phase); (b) Top enriched KEGG pathways in the F3 vs. F2 comparison; (c) GO enrichment bar plot of DEGs in the F5 vs. F3 comparison (initiation of mixed nutrition); (d) Top enriched KEGG pathways in the F5 vs. F3 comparison; (e) GO enrichment bar plot of DEGs in the F9 vs. F5 comparison (transition to full exogenous feeding); (f) Top enriched KEGG pathways in the F9 vs. F5 comparison; (g) GO enrichment bar plot of DEGs in the F14 vs. F9 comparison (exogenous stabilization phase); (h) Top enriched KEGG pathways in the F14 vs. F9 comparison.
**Supplementary Figure 2:** GO and KEGG enrichment analysis of DEGs in the starvation group. (a) GO enrichment bar plot of DEGs for S5 vs. F3; (b) bubble plot showing the top enriched KEGG pathways for S5 vs. F3; (c) GO enrichment bar plot of DEGs for S9 vs. S5; (d) bubble plot showing the top enriched KEGG pathways for S9 vs. S5; (e) GO enrichment bar plot of DEGs for S14 vs. S9; (f) bubble plot showing the top enriched KEGG pathways for S14 vs. S9.
**Supplementary Figure 3:** Comparative functional enrichment analysis of DEGs between starvation (S) and normal (F) groups at synchronous developmental time points. (a) GO enrichment bar plot of DEGs for S5 vs. F5; (b) bubble plot displaying the top enriched KEGG pathways for S5 vs. F5; (c) GO enrichment bar plot of DEGs for S9 vs. F9; (d) bubble plot displaying the top enriched KEGG pathways for S9 vs. F9; (e) GO enrichment bar plot of DEGs for S14 vs. F14; (f) bubble plot displaying the top enriched KEGG pathways for S14 vs. F14.

## Data Availability

The raw transcriptomic sequencing data generated in this study have been deposited in the National Center for Biotechnology Information (NCBI) Sequence Read Archive (SRA) database under the BioProject accession number PRJNA1445138.
