# Supplementary material for: Transcriptomic Indicators of the Point of No Return in Leiocassis longirostris: Ecological Thresholds and Molecular Signatures of Failed Nutritional Transition
Source: Ecol Evol. 2026 Nov 14;16(11):e74726. doi: 10.1002/ece3.74726 (PMC13768842; doi:10.1002/ece3.74726)
Supplement: Supplementary file 1 [file ECE3-16-e74726-s001.docx]

**Supplementary Information (SI)**

**Transcriptomic Indicators of the Point of No Return in *Leiocassis longirostris*: Ecological Thresholds and Molecular Signatures of Failed Nutritional Transition**

**This file includes:**

**Supplementary tables**

Tables 1 to 6

**Supplementary figures**

Figures 1 to 3

**Supplementary tables**

**Supplementary Table 1** Primer sequences used for qRT-PCR validation in *Leiocassis longirostris*

| **Primername** | **sequence（5'to3'）** |
| --- | --- |
| rpl35-F | CTGCGTGGTAAGAAGAAGGAGG |
| rpl35-R | GACAGTAAGAACTCTGGCAATGGA |
| ndufa1-F | GCCTGGATTTGCTATAATGACG |
| ndufa1-R | CTCTTGCGATTCTTTTCTCCTTTC |
| socs3a-F | TGGAACTGTGCCAACCTTATTT |
| socs3a-R | GAGCATCATACGCCTTCAGAAAC |
| nfkbiaa-F | AGCCTGGAAGTGCGAAGTGA |
| nfkbiaa-R | TGGGAGTTGAGGAAGGGGTC |
| stat3-F | TGAAAGACAGCCGTGTATGCC |
| stat3-R | CAGGATGTTGAACTTGCGTGAT |
| traf3-F | GTTTATCTGAACGGTGACGGC |
| traf3-R | AGGACCCTGGTCCATTAGCA |
| rela-F | GCAGAGCAGGCTTCGAGAAA |
| rela-R | CACGGGTGTAGCGGATTGTA |
| Actin-F | GCTACAGCTTCACTACCACA |
| Actin-R | GCCAATGGTGATGACCTGTC |

**Supplementary Table 2** Summary of RNA sequencing quality control metrics for all samples.

| **Samples** | **Clean reads** | **Clean bases** | **GC Content** | **%≥Q30** |
| --- | --- | --- | --- | --- |
| F14R1 | 21,839,593 | 6,551,877,900 | 50.07% | 97.04% |
| F14R2 | 23,887,406 | 7,166,221,800 | 49.19% | 97.13% |
| F14R3 | 42,258,003 | 12,677,400,900 | 50.09% | 97.45% |
| F2R1 | 21,717,508 | 6,515,252,400 | 48.37% | 96.99% |
| F2R2 | 21,699,276 | 6,509,782,800 | 48.25% | 97.08% |
| F2R3 | 22,185,752 | 6,655,725,600 | 48.54% | 96.99% |
| F3R1 | 20,259,523 | 6,077,856,900 | 48.37% | 97.13% |
| F3R2 | 24,717,353 | 7,415,205,900 | 48.54% | 97.66% |
| F3R3 | 20,361,789 | 6,108,536,700 | 47.89% | 97.23% |
| F5R1 | 51,359,866 | 15,407,959,800 | 48.90% | 97.43% |
| F5R2 | 26,968,540 | 8,090,562,000 | 48.57% | 97.32% |
| F5R3 | 30,219,038 | 9,065,711,400 | 49.26% | 97.27% |
| F9R1 | 22,550,344 | 6,765,103,200 | 48.69% | 97.27% |
| F9R2 | 21,997,766 | 6,599,329,800 | 48.94% | 97.13% |
| F9R3 | 22,055,964 | 6,616,789,200 | 49.53% | 97.13% |
| S14R1 | 23,513,078 | 7,053,923,400 | 47.77% | 96.97% |
| S14R2 | 21,817,375 | 6,545,212,500 | 47.60% | 96.87% |
| S14R3 | 54,050,253 | 16,215,075,900 | 47.00% | 97.26% |
| S5R1 | 24,228,278 | 7,268,483,400 | 48.20% | 97.21% |
| S5R2 | 21,906,075 | 6,571,822,500 | 48.47% | 97.18% |
| S5R3 | 21,931,357 | 6,579,407,100 | 48.70% | 97.18% |
| S9R1 | 22,040,343 | 6,612,102,900 | 48.58% | 97.04% |
| S9R2 | 22,081,556 | 6,624,466,800 | 48.69% | 97.00% |
| S9R3 | 22,031,558 | 6,609,467,400 | 48.70% | 97.03% |

**Supplementary Table 3** Number of DEGs at each stage of nutritional transition in the normal group

| **comparable group** | **DEGs_total** | **DEGs_up** | **DEGs_down** |
| --- | --- | --- | --- |
| F3 vs F2 | 211 | 186 | 25 |
| F5 vs F3 | 1058 | 700 | 358 |
| F9 vs F5 | 1354 | 823 | 531 |
| F14 vs F9 | 1368 | 574 | 794 |

**Supplementary Table 4** Number of DEGs at each stage of stress escalation in the starvation group

| **comparable group** | **DEGs_total** | **DEGs_up** | **DEGs_down** |
| --- | --- | --- | --- |
| S5 vs F3 | 685 | 433 | 252 |
| S9 vs S5 | 1976 | 998 | 978 |
| S14 vs S9 | 7087 | 3449 | 3638 |

**Supplementary Table 5** The top 15 core regulatory genes

| **Functional Family** | **Gene Name** |
| --- | --- |
| NF-κB Family | *Llo_GLEAN_10021876、Llo_GLEAN_10016938* |
| IκB Family | *Llo_GLEAN_10020298、Llo_GLEAN_10011184* |
| TRAF Family | *Llo_GLEAN_10011394* |
| STAT Family | *Llo_GLEAN_10006023、Llo_GLEAN_10005352* |
| TNF/TNFR Superfamily | *Llo_GLEAN_10009413、Llo_GLEAN_10005765* |
| Inflammatory Factor Family | *Llo_GLEAN_10007098、Llo_GLEAN_10007099* |
| Bcl-2 Family | *Llo_GLEAN_10005564* |
| IAP Family | *Llo_GLEAN_10008227* |
| Kinase Family | *Llo_GLEAN_10022682* |
| RTK Family | *Llo_GLEAN_10023544* |

**Supplementary Table 6** Statistical comparison of the quantity of DEGs between the normal groups and the starvation groups within the same period.

| **comparable group** | **DEGs_total** | **DEGs_up** | **DEGs_down** |
| --- | --- | --- | --- |
| S5 vs F5 | 240 | 85 | 155 |
| S9 vs F9 | 4550 | 2106 | 2444 |
| S14 vs F14 | 8897 | 4462 | 4435 |

**Supplementary figures**

**Supplementary Fig. 1.** Longitudinal functional enrichment analysis of DEGs during nutritional transition.**a** GO enrichment bar plot of DEGs in the F3 vs. F2 comparison (pure endogenous phase); **b** Top enriched KEGG pathways in the F3 vs. F2 comparison; **c** GO enrichment bar plot of DEGs in the F5 vs. F3 comparison (initiation of mixed nutrition); **d** Top enriched KEGG pathways in the F5 vs. F3 comparison; **e** GO enrichment bar plot of DEGs in the F9 vs. F5 comparison (transition to full exogenous feeding); **f** Top enriched KEGG pathways in the F9 vs. F5 comparison; **g** GO enrichment bar plot of DEGs in the F14 vs. F9 comparison (exogenous stabilization phase); **h** Top enriched KEGG pathways in the F14 vs. F9 comparison.

**Supplementary Fig. 2.** GO and KEGG enrichment analysis of DEGs in the starvation group. **a** GO enrichment bar plot of DEGs for S5 vs. F3; **b** bubble plot showing the top enriched KEGG pathways for S5 vs. F3; **c** GO enrichment bar plot of DEGs for S9 vs. S5; **d** bubble plot showing the top enriched KEGG pathways for S9 vs. S5; **e** GO enrichment bar plot of DEGs for S14 vs. S9; **f** bubble plot showing the top enriched KEGG pathways for S14 vs. S9.

**Supplementary Fig. 3.** Comparative functional enrichment analysis of DEGs between starvation (S) and normal (F) groups at synchronous developmental time points. **a** GO enrichment bar plot of DEGs for S5 vs. F5; **b** bubble plot displaying the top enriched KEGG pathways for S5 vs. F5; **c** GO enrichment bar plot of DEGs for S9 vs. F9; **d** bubble plot displaying the top enriched KEGG pathways for S9 vs. F9; **e** GO enrichment bar plot of DEGs for S14 vs. F14; **f** bubble plot displaying the top enriched KEGG pathways for S14 vs. F14.
